# CELF1 promotes matrix metalloproteinases gene expression at transcriptional level in lens epithelial cells

**DOI:** 10.1186/s12886-022-02344-8

**Published:** 2022-03-14

**Authors:** Jun Xiao, Xin Tian, Siyan Jin, Yanhui He, Meijiao Song, He Zou

**Affiliations:** 1 Department of Ophthalmology, The Second Hospital of Jilin University, Changchun city, Jilin province China; 2 Department of Pediatrics, The Second Hospital of Jilin University, Changchun city, Jilin province China

**Keywords:** CELF1, Transcriptional regulation, RNA-seq, MMPs, Cataract

## Abstract

**Background:**

RNA binding proteins (RBPs)-mediated regulation plays important roles in many eye diseases, including the canonical RBP CELF1 in cataract. While the definite molecular regulatory mechanisms of CELF1 on cataract still remain elusive.

**Methods:**

In this study, we overexpressed CELF1 in human cultured lens epithelial SRA01/04 cells and applied whole transcriptome sequencing (RNA-seq) method to analyze the global differences mediated by CELF1. We then analyzed public RNA-seq and CELF1-RNA interactome data to decipher the underlying mechanisms.

**Results:**

The results showed that transcriptome profile was globally changed by CELF1 overexpression (CELF1-OE). Functional analysis revealed CELF1 specifically increased the expression of genes in extracellular matrix disassembly, extracellular matrix organization, and proteolysis, which could be classified into matrix metalloproteinases (MMPs) family. This finding was also validated by RT-qPCR and public mouse early embryonic lens data. Integrating analysis with public CELF1-RNA interactome data revealed that no obvious CELF1-binding peak was found on the transcripts of these genes, indicating an indirectly regulatory role of CELF1 in lens epithelial cells.

**Conclusions:**

Our study demonstrated that CELF1-OE promotes transcriptional level of MMP genes; and this regulation may be completed by other ways except for binding to RNA targets. These results suggest that CELF1-OE is implicated in the development of lens, which is associated with cataract and expands our understanding of CELF1 regulatory roles as an RNA binding protein.

**Supplementary Information:**

The online version contains supplementary material available at 10.1186/s12886-022-02344-8.

## Background

Cataract, characterized by opacified lens, is the most common cause of reversible loss of vision which troubled nearly 24 million people worldwide [1]. White reflex of eye is an important symptom of cataract, named Leukocoria [2]. Lens is a transparent biconvex structure in eye that maintains the eye clarity and focus light onto the retina. It is composed of fibers, which are generated from the lens epithelium and then migrate from the periphery towards the center. Under normal circumstances, newly formed lens cells adhere externally to older cells. However, once epithelial cells are unable to shed so that differentiate into lens fibers, they will plie up centrally, with the oldest cells being in the center of the lens, ultimately develop into cataract with discoloration and opacities in the lens [1]. Drugs, chemical injury, mechanical trauma, ionising, infrared and ultraviolet radiate are common risk factors contributing to cataractogenesis [3].

RNA-binding proteins (RBPs) play an essential role in the process of post-transcriptional regulation. RNAs interact with RBPs through a series of canonical RNA-binding domains to form ribonucleoprotein complexes that involved in many post-transcriptional events including mRNA stability, polyadenylation, splicing, localization, and degradation [4, 5]. The importance of RBPs in eye development and diseases has been received with concern in recent years [6]. RBPs are also important for lens development and cataract pathogenesis. For example, TDRD7 was firstly identified to be involved in cataract in human [7]. Caprin2 is another RBP that causes severe lens defects and features of Peter Anomaly in Caprin2-knockout mouse [8]. More recently, CELF1 was also identified as an important RBP in regulating post-transcription during lens development [9]. The expression of CELF1 was high and enriched in lens during the embryonic development of mice [10], suggesting its potential regulatory roles in lens development and cataractogenesis.

As a multifunctional RBP, CELF1 was known to preferentially bind to GU-rich elements (GREs) predominantly located in 3’ untranslated regions (UTRs) of target mRNAs [11]. Based on this structure, CELF1 has been implicated in various post-transcriptional processes, such as alternative splicing [12], localization [13], decay [14], and translation [15]. RNA binding sites of CELF1 was also identified by CLIP-seq, which promotes to characterize the genome-wide functions of CELF1 in human HeLa cells [16]. Regarding to the important roles of CELF1 in diverse biological processes, CELF1 dysregulation usually resulted in many diseases. Precocious myotube formation in mouse myoblasts is caused by knockdown of Celf1, which may play a negative role in terminal myocyte differentiation [17]. In type 1 diabetic mouse hearts, mutations of CELF1 binding sites impair alternative splicing regulation by CELF1, leading to abnormal gene expression [18]. Previous studies have also shown that CELF1 was associated with cataract by functioning as an RNA binding protein. Severe eye defects and cataract were presented in Celf1-knockout mice or Celf1-knockdown zebrafish and Xenopus [9]. To achieve lens transparency, Celf1 regulates key factors, such as p27^Kip1^ and Dnase2b which are necessary for degradation of nuclear and fiber cell morphology by controlling the expression level of target mRNAs, providing new Celf1-regulated mechanisms involved in lens transparency. Using immunofluorescence assays, abnormally high expression of Pax6 protein was observed in CELF1 knockdown lenses compared to control lenes, suggesting that the eye transcription factor PAX6 is a key target regulated by CELF1 in lens development [19]. In addition, Aryal et al [20] found that Celf1 could post-transcriptionally control the spatiotemporal expression of the key homeodomain transcription factors Pax6 and Prox1 in the lens development in mice. RNA-immunoprecipitation assays showed that Celf1 negatively controls Pax6 and Prox1 translation through binding to their 3’ UTRs. These findings indicated that Celf1 plays a crucial role in lens development, and that it is a potential candidate for therapeutic treatment of cataract.

However, the exact mechanism of gene expression level regulation mediated by CELF1 during lens development or cataractogenesis is largely unknown. To gain insight into the underlying molecular mechanisms, we obtained transcriptome profile after CELF1 overexpression in human lens epithelial SAR01/04 cells, and made a comprehensive analysis of the RNA-seq data with the public datasets. Our results revealed that a large number of genes in proteolysis were increased upon CELF1-overexpression; these genes were primarily MMPs, which were associated with cataract formation. Our findings expanded the understanding of critical CELF1 functions in lens development and provided a potential link with cataractogenesis.

## Materials and methods

### Cell culture and transfection

SRA01/04 cells, a lens epithelial cell line widely used in lens study, were obtained from the Institute of Biochemistry and Cell Biology (Chinese Academy of Science, Shanghai, China) and cultured in DMEM with 10% fetal bovine serum (FBS), penicillin (100 U/μl) and streptomycin (100 μg/ml). To increase the expression level of CELF1, a CELF1-overexpressing (CELF1-OE) plasmid was transfected into SRA01/04 using Lipofectamine 2000 based on the manufacturer’s protocol. SRA01/04 cells transfected with empty vector (Control) were used as control. After 48 h, CELF1-OE and control cells were collected for RT-qPCR experiment.

### Assessment of CELF1-overexpression

To assess the efficiency of CELF1-overexpression, cDNA synthesis was conducted according to standard procedures followed by RT-qPCR, with the expression level of GAPDH as control. Transcript levels of CELF1 were measured by comparing with GADPH expression using 2^-ΔΔCT^ method [21].

### Western blot analysis

After transfection for 48 h, SRA01/04 were grouped and lysed in RIPA buffer, the samples were centrifuged at a speed of 12,000 rpm for 5min, supernatants were heated at 100 °C for 10 min. The samples were subjected to SDS-PAGE and subsequently transferred onto PVDF membranes. After blocking with 5% skim milk for one hour, the membranes were incubated with monoclonal Flag antibody (1:1,000 dilution; polyclonal antibody; cat. no.2368S; CST) at 4 °C overnight and GADPH as control (1:2000 dilution; polyclonal antibody; cat. no. A19056; ABClonal). Then the membranes were incubated with secondary antibody for an hour. Finally, the membranes were visualized by enhanced chemiluminescence (ECL).

### Complementary DNA (cDNA) library preparation and RNA-seq

Total RNA was extracted by Trizol and further purified with two phenol-chloroform treatments, to acquire purified RNA, RQ1 DNase was added to remove DNA. Further, absorbance at 260 nm/280 nm was detected by Agilent Bioanalyzer 2100 to assess the quantity and quality of RNA.

Before RNA-sequencing, 10 μg polyadenylated mRNA were prepared and concentrated with oligo (DT)-conjugated magnetic beads for each sample. Then, the mRNA samples were iron fragmented at 95 °C, followed by end repair, A tailing, and reverse transcribed with RT primer harboring 3’ adaptor sequence and randomized hexamer. Finally, the cDNAs were amplified for sequencing.

To insure high-quality reads, several criteria were set as following: Firstly, raw reads that were more than 2-N bases were abandoned; Secondly, filtered low quality bases and adaptors from raw reads; Thirdly, short reads that were less than 16nt were also removed; At last, clean reads were mapped to the GRch38 genome by TopHat2 [22]. The libraries were applied to NextSeq 500 system for 150 nt paired-end sequencing (ABLife Inc., Wuhan).

### Bioinformatics analysis

Fragments per Kilobase per Million (FPKM) was used to evaluate gene expression level [23]. Meanwhile, R package edgeR [22] was applied to filter out differentially expressed genes (DEGs), fold change (FC ≥ 1.5) and false discovery rate (FDR < 0,05) were set as criteria of DEGs.

In addition, Gene Ontology (GO) terms and KEGG pathways were carried out to sort out functional categories of DEGs [24]. Hypergeometric test and Benjamini-Hochberg FDR controlling procedure were used to define the enrichment of each pathway. We used Find Individual Motif Occurrences (FIMO) software [25] to search CELF1-bound UG-rich motif within the 3’UTR region of DEGs with default parameters.

### Validation of DEGs by qPCR

To elucidate the validity of our RNA-seq data, quantitative real-time PCR was performed in several candidate genes. PCR amplifications were performed in triplicate for each sample with conditions consisting of denaturing at 95 °C for 10 min, 40 cycles of denaturing at 95 °C for 15 s, annealing and following by extension at 60 °C for 1 min.

To explore the mRNA binding profile of CELF1, we obtained and analyzed the RNA ligands and binding sites of CELF1 in HeLa cells from two sets of published data SRP0935 and PRJEB12208 [12, 16]. Furthermore, we also analyzed correlation between CELF1 and candidate DEGs in house mouse of different embryonic lens development from published data GSE119596 [10], in which the total number and expression level of cataract or lens defects-related genes, including CELF1 progressively increased as the lens develops from E10.5 through E16.5 stages. It implies that older stages might exhibit higher risks of cataract formation than that of younger stages.

### Statistical analysis

For statistical method, all values were presented with mean ± standard deviation, SPSS statistical software (Chicago, IL) was used to analyze data. Only if the *p*-value was less than 0.05 (*p* < 0.05) then the difference was considered significant.

## Results

### Assessment of CELF1-overexpression in SRA01/04 cells

**Fig. 1:**
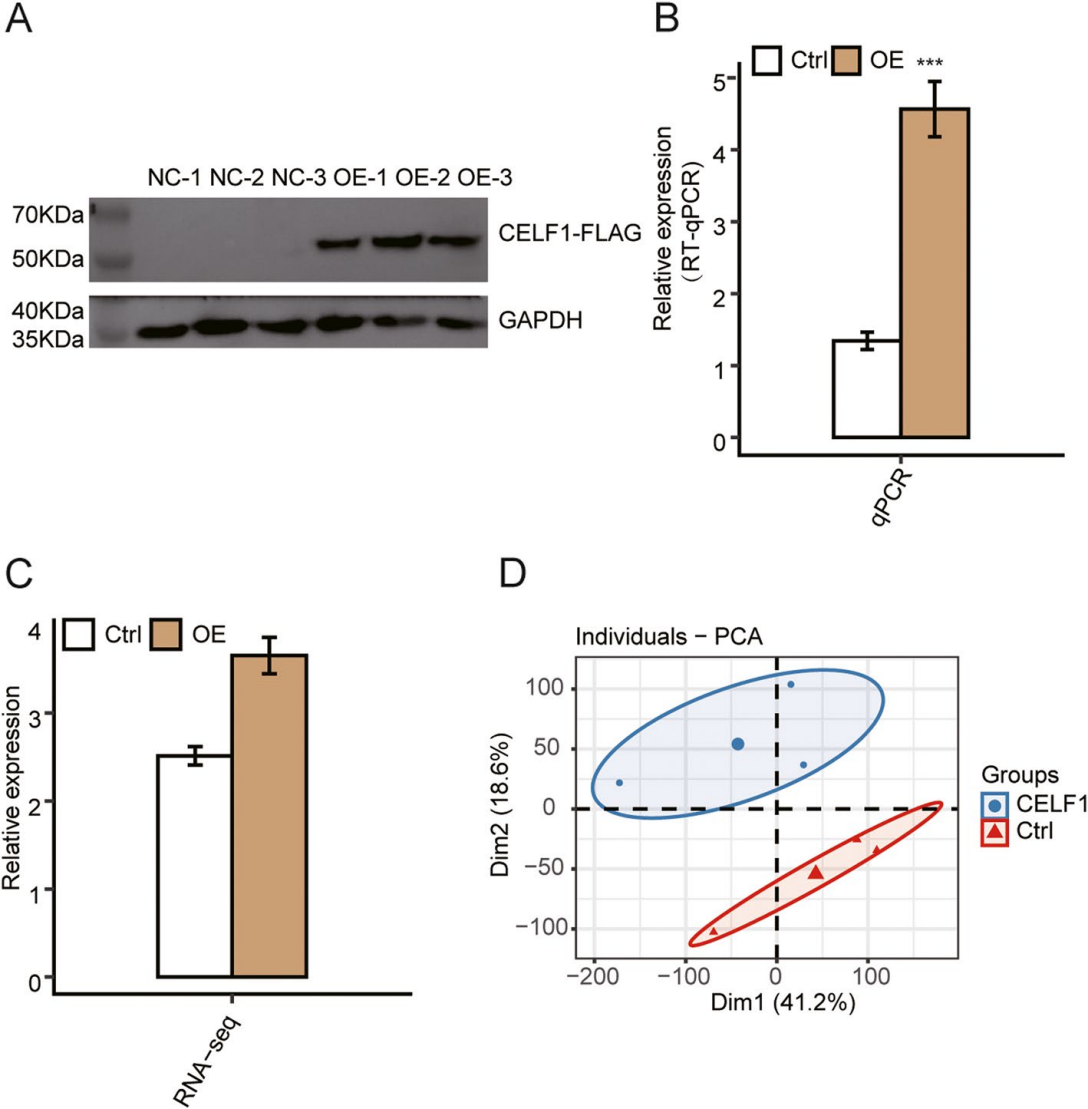
Assessment of CELF1-overxpression in SRA01/04 cells. **A** Validation of CELF1-overexpression by Western Blot. **B** Validation of CELF1-overexpression by qPCR. **C** The FPKM values of CELF1 were calculated in RNA-seq. **D** Principal component analysis of all expressed genes between the control and CELF1 overexpression samples. Data are represented as the mean ± standard deviation. Student’s test was performed to compare CELF1-overexpression SRA01/04 cells and control with significance set at a *p* value of less than 0.05. *P < 0.05, ***P < 0.001

To comprehensively investigate CELF1-mediated transcriptional regulation, SRA01/04 cells were transfected with a *CELF1*-overexpressing (OE) plasmid using Lipofectamine 2000 followed by whole transcriptome sequencing (RNA-seq). We constructed six RNA-seq libraries and sequenced for CELF1-overexpression and control SRA01/04 cells, with three replicates for each group. The protein level of CELF1 was increased in CELF1-OE samples compared with control samples, as assessed by western blot in Fig. 1A. Accordingly, the mRNA level of CELF1 was increased approximately 4 times compared with control by RT-qPCR (Fig. 1B). Further, the efficiency of CELF1-overexpression was also confirmed by RNA-seq analysis (Fig. 1C), indicating that overexpression of CELF1 in SRA01/04 cells was successful. FPKM values were applied for calculating the variation contribution of principal components between CELF1-overexpression and control cells, the distribution of principal component analysis (PCA) showed that the biological replicates were clustered together by the second component (Fig. 1D).

### CELF1 overexpression regulates transcription in SRA014/01 cells

After obtaining the raw RNA-seq reads, we discarded the adaptor sequences and low-quality reads, generating a total of 77.6 ± 5.4 million raw reads per sample and 74.3 ± 5.8 million clean reads per sample (Table 1), in which an average of 72.0 ± 5.2 million were paired-end reads. Based on the mapping results by TopHat2 [22], 95.59-96.72 % of those paired-end reads were mapped to the human GRCH38 genome, and about 85.74-96.95 % were uniquely aligned (Table 1). These results supported the validity of sequencing data in our experiments. To identify the gene expression profiles regulated by CELF1, FPKM values were applied to calculate the expression level of candidate genes. RNA-seq yielded 19,703 genes, in which 9,585 genes were detected at an expression level of FPKM>1 in at least one sample.

**Table 1:** Information of RNA-seq reads in experiment

Sample	CELF1_SRA_1st	CELF1_SRA_2nd	CELF1_SRA_3rd	Ctrl_1st	Ctrl_2nd	Ctrl_3rd	Mean
Raw reads	79902626	84904120	81594846	70549626	75180916	73338412	77578424.33±5446634.377
Clean reads	77004105	81761685	78505488	66213178	71926994	70345341	74292798.5 ±5785186.607
Paired-end reads	74701846	79255492	76261510	64028722	69738738	68075308	72010269.33±5198713.892
Total mapped(%)	71677060(95.95%)	76091134(96.01%)	73420345(96.27%)	61822687(96.55%)	67454303(96.72%)	65835489(96.71%)	69383503±4830889.588
Uniquely mapped(%)	63361981(88.4%)	68826896(90.45%)	62950881(85.74%)	57030340(92.25%)	63156202(93.63%)	63830263(96.95%)	63192760.5±3420862.379
Splice reads (%)	32010772(50.52%)	35210711(51.16%)	33379884(53.03%)	29918008(52.46%)	32659050(51.71%)	33157280(51.95%)	32722617.5±1742879.187

**Fig. 2:**
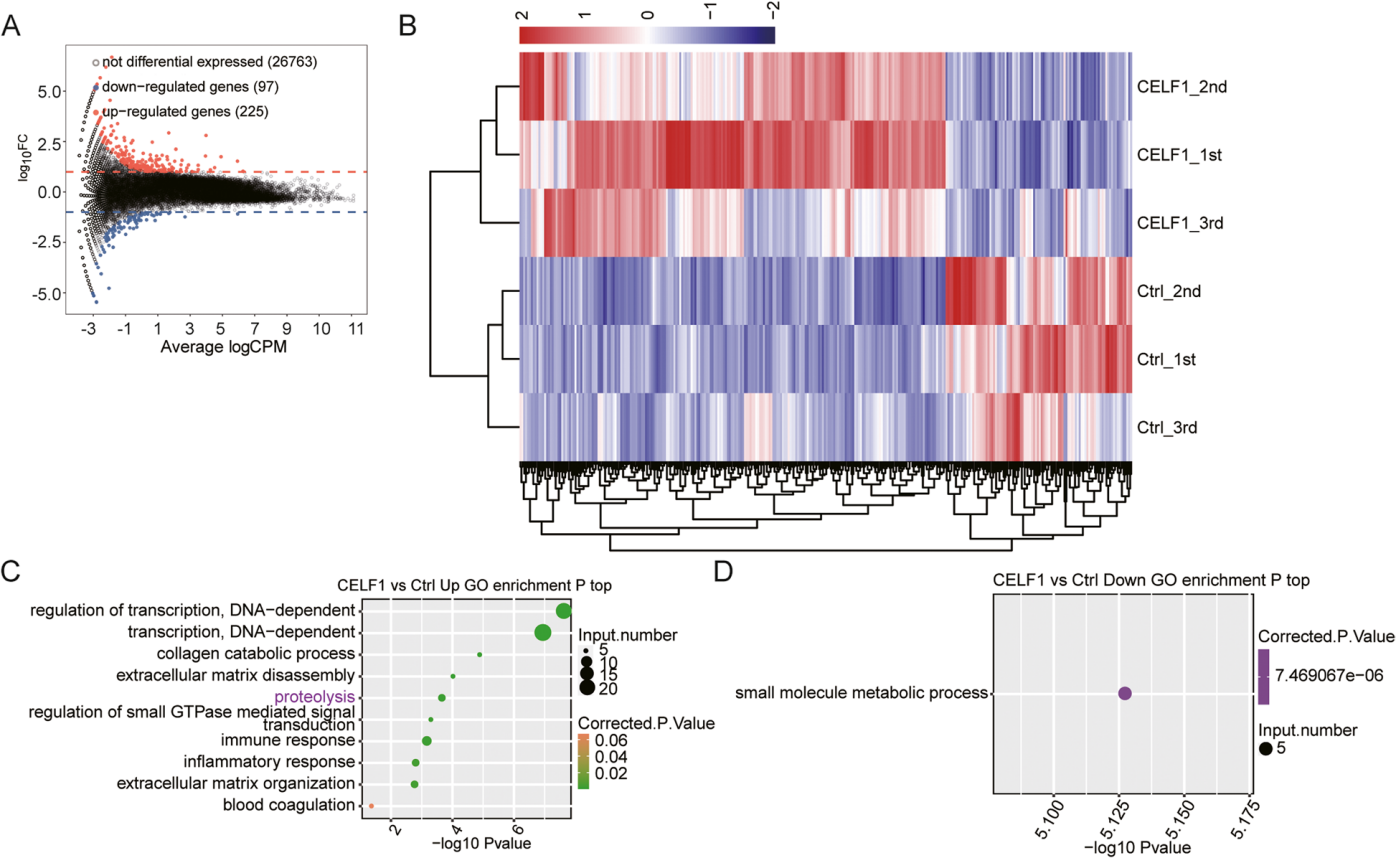
RNA-seq analysis of CELF1 regulated transcriptome in SRA01/04 cells. **A** Identification of CELF1 regulated genes. Red dots indicated up-regulated genes, whereas blue dots indicated down-regulated genes. **B** Heatmap of all DEGs in control and CELF1 overexpression samples. The most representative GO biological processes of up-regulated genes (**C**) and down-regulated genes (**D**)

To analyze genes responding to elevated CELF1 in SRA01/04 at the whole transcriptome level, edgeR [26] was used to perform differentially expressed genes (DEGs) analysis on our data. When we set the cut-off as fold change ≥ 2 or ≤ 0.5 and a 5% false discovery rate (FDR), 97 down-regulated and 225 up-regulated genes were identified, respectively (Fig. 2A; Table S1). The results demonstrated that CELF1 significantly changed gene expression profile in SRA01/04 cells. Hierarchical clustering heat map analysis of DEGs expression pattern successfully and clearly separated CELF1-OE and control samples, revealing a high consistency among the triplicates data sets (Fig. 2B).

To further characterize the potential functions of these DEGs, all the identified DEGs were annotated by GO and KEGG functional enrichment analysis. The results revealed that upregulated DEGs were significantly enriched in GO biological process pathways, which were primarily associated with regulation of transcription, collagen catabolic process, extracellular matrix (ECM) disassembly, proteolysis, and extracellular matrix organization (Fig. 2C). ECM and proteolysis terms contained several common genes, including MMP9, MMP7, CTSS, and MMP1. The top two GO BP pathways were regulation of transcription related pathways, suggesting that CELF1 could promote expression level of transcription regulators. While downregulated DEGs were only enriched in one GO terms: Small molecule metabolic process (Fig. 2D). When we set *p*-value as < 0.05 for KEGG pathways, we analyzed and presented the top ten KEGG pathways for upregulated DEGs (Fig. S1A), including cytokine-cytokine receptor interaction, chemokine signaling pathway and apoptosis. Whereas, down-regulated DEGs were enriched primarily in fat digestion and absorption and pathogenic *Escherichia coli* infection (Fig. S1B).

### Elevated CELF1 modulates expression of genes involved in proteolysis

**Fig. 3:**
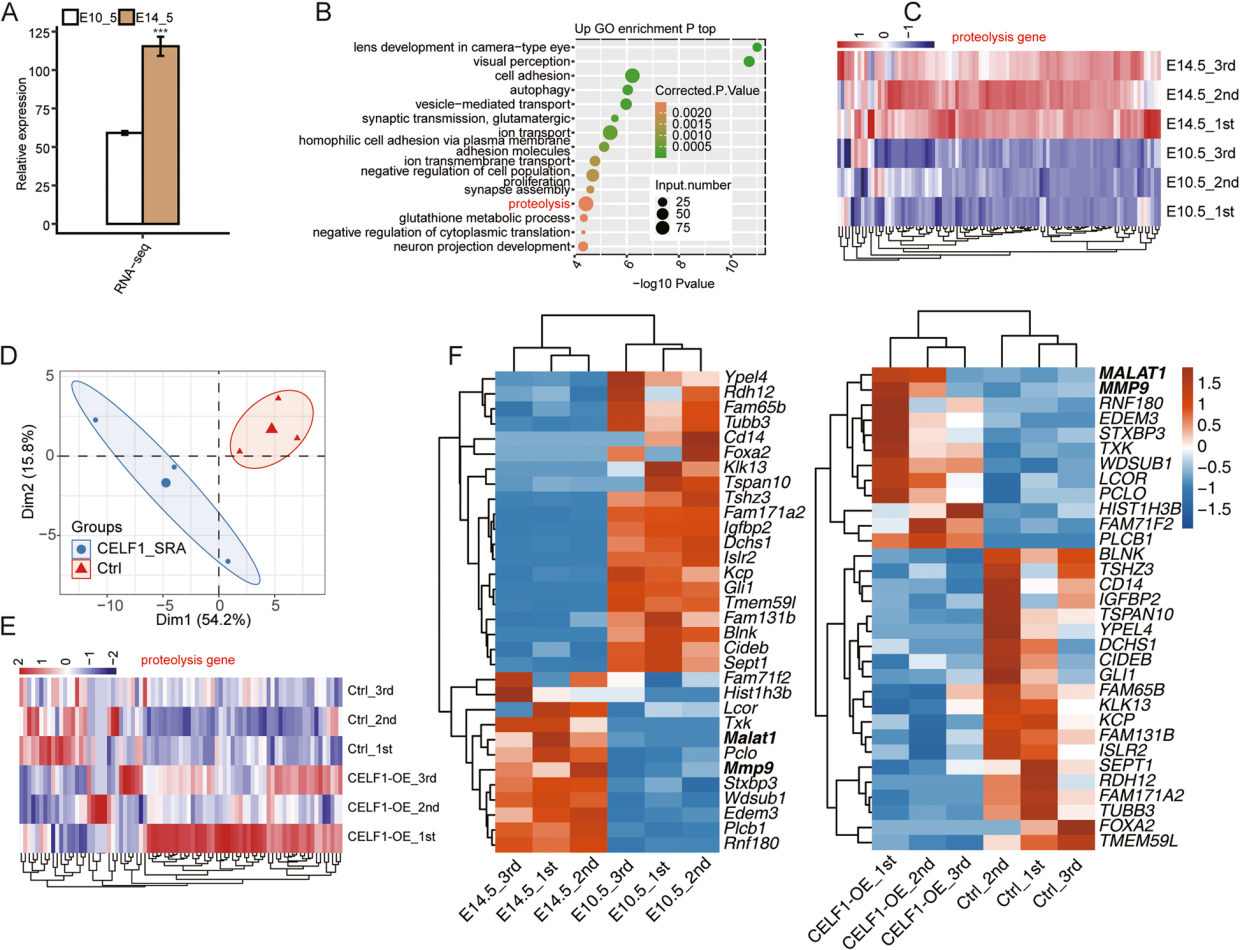
Elevated CELF1 extensively regulates expression of genes involved in proteolysis. **A** Relative expression level of CELF1 determined by RNA-seq between E10.5 and E14.5 in mouse lens. **B** The top 15 representative GO biological processes of up-DEGs between E10.5 and E14.5 in mouse lens. **C** Hierarchical clustering heatmap of up-DEGs from proteolysis term in (**B**). **D** PCA result showed the expression pattern of proteolysis-related genes in CELF1-OE and control samples from this study. **E** Hierarchical clustering heatmap of proteolysis-related genes in CELF1-OE and control samples from this study. **F** Hierarchical clustering heatmap showed the expression pattern of overlapped DEGs in E10.5 and E14.5 in mouse lens (left panel) and CELF1-OE and control samples from this study (right panel)

To further confirm the reliability of the RNA-seq results, we downloaded a set of RNA-seq data (GSE119596) from early embryonic lens of mouse [10], in which the total number and expression level of contact or lens defects-related genes progressively increased as the lens developed from lens pit stage (embryonic day (E) 10.5) through secondary fiber differentiation stage (E16.5), implying that the embryonic lens of mouse in older stages might exhibit higher risks of cataract than that of younger stages. More important, a significant elevated Celf1 was observed when early embryonic lens developed from E10.5 to E14.5 (Fig. 3A), indicating that elevated Celf1 might associated with lens formation and development in mouse. Based on the elevated expression level of Celf1, the transcriptome profile of embryonic lens both in E10.5 and E14.5 could be a substitute of CELF1-OE dataset and were selected for further analysis to make a comparison with our data. A total of 7555 DEGs were identified from embryonic lens RNA-seq data. GO biological process enrichment analysis revealed that up-DEGs in E14.5 samples were enriched in lens development in camera-type eye, visual perception, cell adhesion, autophagy, and proteolysis, indicating elevated CELF1 may participate in regulating expression of genes implicated in lens development (Fig. 3B). In addition, enriched proteolysis term was also emerged in the top 10 representative GO BP terms of up-DEGs in CELF1-OE SRA01/04 cells (Fig. 2C). Thus, a heat map was generated to compare expression profile of genes enriched in proteolysis. Hierarchical clustering of normalized FPKM values of DEGs involved in proteolysis pathway showed a clear separation of proteolysis-related genes was observed between E10.5 and E14.5 (Fig. 3C). We then analyzed the expression pattern of proteolysis related genes in our dataset. PCA result of these genes also showed a clear separation between CELF1-OE and control cells (Fig. 3D). Hierarchical clustering heatmap of these genes in our dataset demonstrated more genes were with higher expression levels in CELF1-OE samples although the consistency is not so good among three replicates (Fig. 3E). Meanwhile, we also identified 12 up-DEGs and 20 down-DEGs overlapped with our CELF1-OE RNA-seq dataset. Among the 12 overlapped up-DEGs, MMP9 and lncRNA-MALAT1 were observed (Fig. 3F). These results suggest that elevated CELF1 could modulate the expression level of genes associated with proteolysis both in SRA01/04 cells and mouse lens at embryonic stages, implying the important role of proteolysis in lens development.

### Further validation of CELF1-regulated gene transcription in proteolysis

**Fig. 4:**
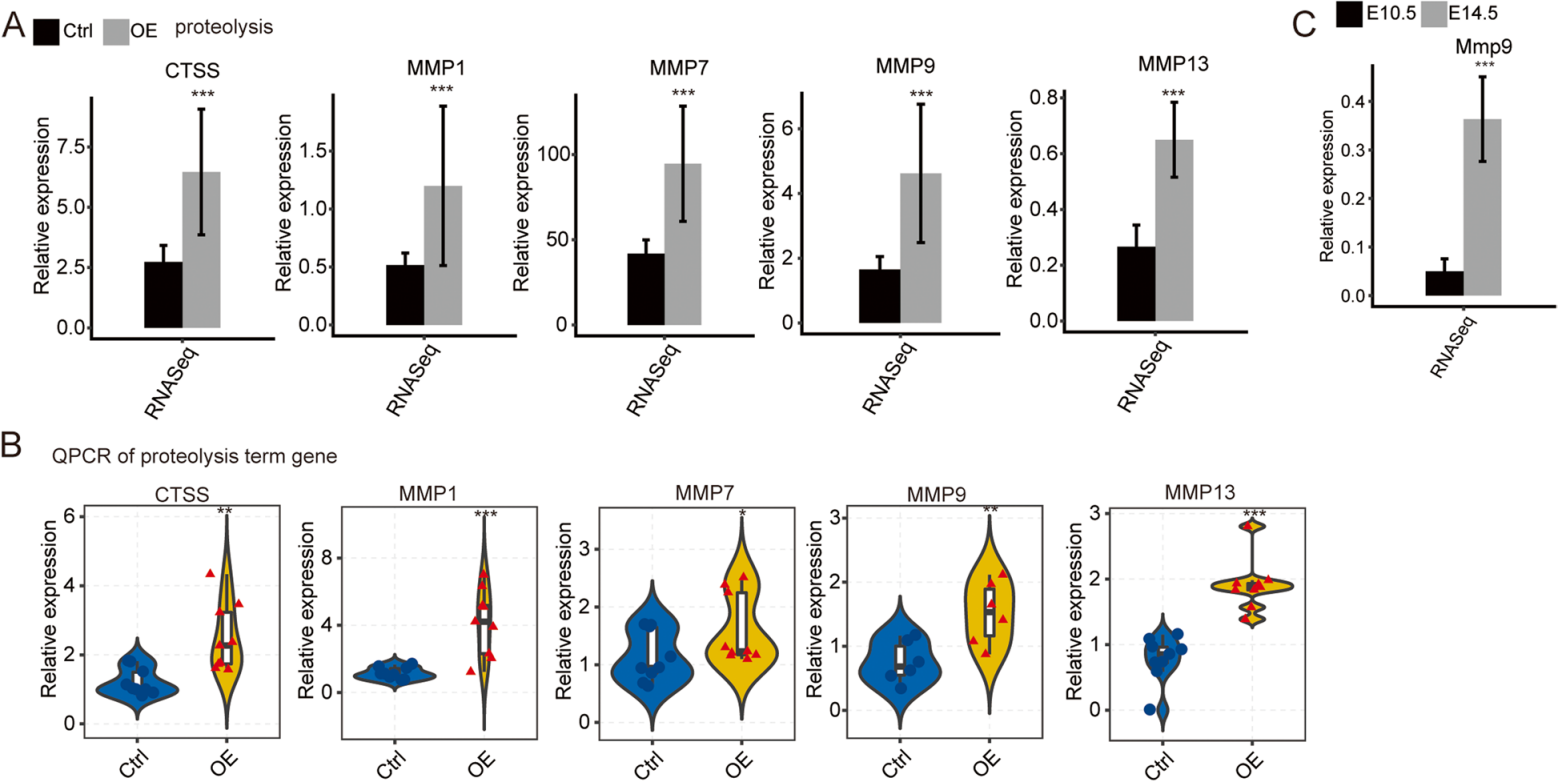
Validation of CELF1-regulated genes. Validation of DEGs in SRA01/04 cells measured by RNA-seq (**A**) and qPCR (**B**). **C** Relative expression levels of MMP9 in mouse lens measured by RNA-seq. For qPCR, GAPDH was used as the reference gene

In order to verify the DEGs results in RNA-seq, we performed RT-qPCR analysis to determine the gene expression levels. Five up-regulated DEGs from proteolysis were selected for RT-qPCR analysis, including MMP1, MMP7, MMP9, MMP13, and CTSS (Fig. 4A). The primers of these genes were shown in Table 2. A statistically significant increase of those genes was observed in RT-qPCR experiment (Fig. 4B), which was in line with the FPKM values (Fig. 4A). These results demonstrated the high correlation between RNA-seq and RT-qPCR results, confirming the discoveries in RNA-seq dataset. Furthermore, we also found that increased CELF1 expression level was coupled with elevated expression of MMP9 in embryonic lens of mouse (Fig. 4C), when embryonic lens developed from E10.5 to E14.5. Collectively, the results suggested that CELF1 may participate in lens development or cataract formation by regulating expression level of genes in proteolysis, particularly, matrix metalloproteinases.

**Table 2:** Primers for detected genes

Primer Name	Sequence 5’ to 3’
MMP1-F	CAGGGACAGAATGTGCTA
MMP1-R	TTTCCAGTGTTTTCCTCA
MMP7-F	AAGCCAAACTCAAGGAGA
MMP7-R	AGTCCATTTTGGGCTATT
MMP9-F	CCTTCTACGGCCACTACT
MMP9-R	ATCCTTGAACAAATACAGC
MMP13-F	TTTTGGGCTCTTAATGGT
MMP13-R	AGTCTTGCCTGTATCCTC
CTSS-F	CCAAGGCAGGCATATCAA
CTSS-R	TGGGTTCAAGGAATCTCG

### Functional analysis of genes bound by CELF1

**Fig. 5:**
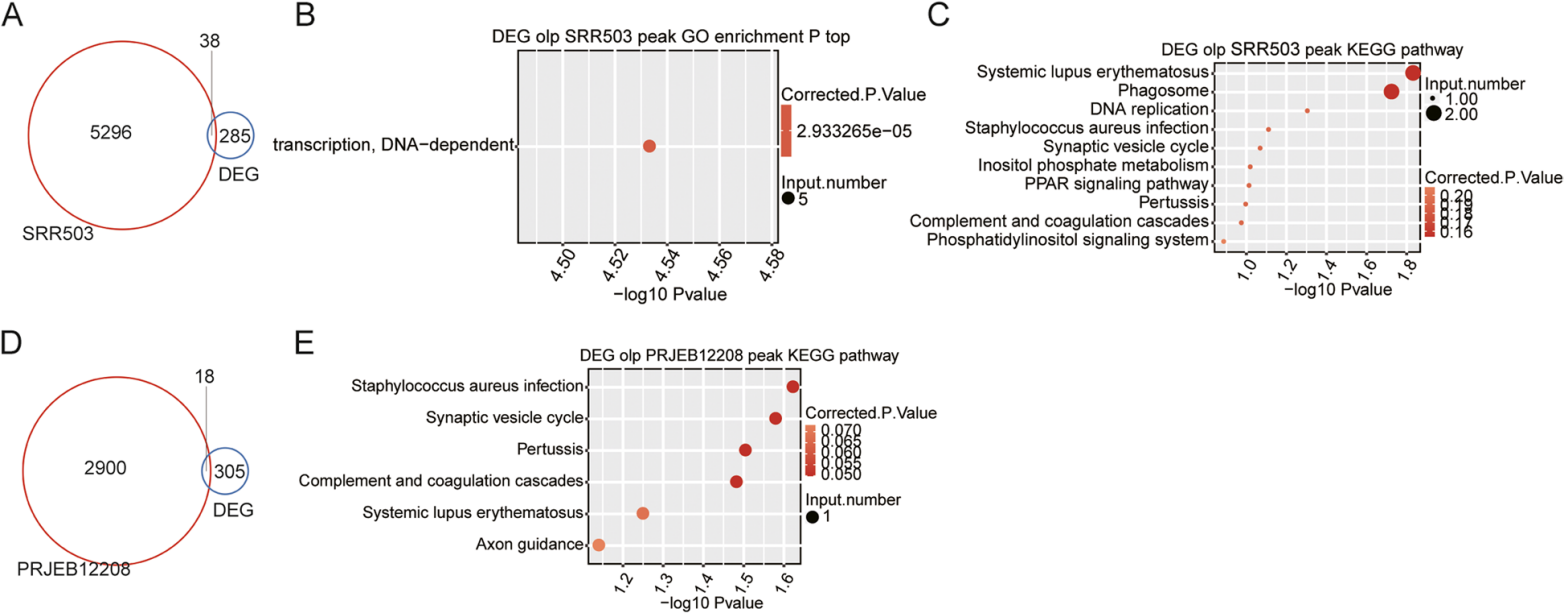
Function analysis of CELF1-bound genes. **A** CELF1-regulated DEGs overlapped with the CELF1-bound genes from SRR503. **B** The most representative GO biological processes of overlapped genes between DEGs and CELF1-bound genes from SRR503. **C** The top representative KEGG pathways of overlapped genes between DEGs and CELF1-bound genes from SRR503. **D** CELF1-regulated DEGs overlapped with the CELF1-bound genes from PRJEB12208. **E** The top representative KEGG pathways of overlapped genes between DEGs and CELF1-bound genes from PRJEB12208

We downloaded a set of CELF1 RIP-seq data [12] and a set of CELF1 CLIP-seq data for HeLa cells [16] to evaluate whether CELF1 directly bind to those genes and subsequently regulate their expression levels. After performing overlapping analysis, we detected 38 DEGs in our study overlapped with CELF1-targeted genes in RIP-seq experiment (Fig. 5A) and only 18 DEGs overlapped in CLIP-seq experiment (Fig. 5D). GO functional enrichment analysis was performed on overlapped DEGs between RIP-seq data and our data, 5 up-regulated DEGs were enriched in transcription, DNA-dependent (Fig. 5B), which is consistent with the GO BP enrichment analysis of DEGs (Fig. 2C). KEGG pathways (*p*-value<0.05) were primarily enriched in Systemic lupus erythematosus, Phagosome, DNA replication, and *Staphylococcus aureus* infection (Fig. 5C). By analyzing the genes validated by RT-qPCR in Fig. 4, we did not find obvious CELF1 peaks that were enriched on the transcripts of MMP1, MMP9, MMP13, or CTSS. We then searched canonical CELF1-bound UG-rich motif [27] in the 3’UTR region of DEGs. The motif probability matrix was shown in Table S2, which was obtained by re-analyzing the CELF1 RIP-seq data [12]. We found 86 DEGs contained UG-rich motif, while DEGs validated in Fig. 4 were not among them. These results indicate that CELF1 might regulate expression of most DEGs by an indirect manner, e.g. via regulation of one or more DNA-binding transcription factors that control their expression levels at the transcriptional level. Alternatively, Celf1 can interact with other RNA-binding proteins associated with these 3’-UTRs [28].

## Discussion

Cataract has been characterized as severe eye disease with opacified lens, and loss of epithelial polarity and cell multi-layering are primary signs of pathogenesis in cataractogenesis. Siddam teams applied a bioinformatics tool iSyTE to identify a novel RNA binding protein, CELF1, which is important for lens development and cataract formation [9]. Their results showed that *Celf1*-knockout in mice, or knockdown in zebrafish or Xenopus morphants resulted in severe eye defects or cataract. Except for TDRD7 and Caprin2 [8, 29, 30], CELF1 was identified as another RBP that plays a crucial role during cataract formation [20]. However, CELF1-overexpression (CELF1-OE) meditated transcriptional or post-transcriptional regulations in lens development or cataract formation remains to be resolved.

To have a deeper understanding of CELF1 functions on lens development and cataract, we performed RNA-seq experiments in CELF1-OE SRA01/04 cells and control cells. Our results showed that elevated CELF1 globally changed expression profile in SRA01/04 cells. As an RNA binding protein, overexpression of CELF1 has been associated with many diseases, for example, CELF1-OE was correlated with lower levels of endogenous p27, at the same time, repressing p27 IRES activity in human breast cancer cell line MCF7 [15]. Moreover, elevated CELF1 expression mediated defects of myocytes with CUG-expansion, by increasing myocyte cycling [17]. In breast epithelial cells, CELF1-OE promoted the translation of epithelial to mesenchymal transition (EMT) and ultimately tumor progression [31]. All together, these findings indicated that CELF1-OE plays an important role in pathological processes of diseases. In agreement with previous studies, we provided evidence that elevated CELF1 may also affect cataract pathogenesis and lens development.

Based on GO analysis results, the up-DEGs by CELF1-OE were highly enriched in transcriptional regulation, extracellular matrix (ECM) disassembly and organization, and proteolysis, which were presented in the top 10 pathways. As we know, protein turnover in ECM is common and important as a fundamental feature of many normal and pathological processes [32]. Undoubtedly, alteration in extracellular matrix turnover is also associated with cataract [33, 34]. Posterior capsule opacification (PCO), which is also known as secondary cataract, was characterized by cellular migration onto the posterior lens capsule, coupling with deposition of abnormal extracellular matrix and capsular wrinkling, all of which could lead to opacification of lens and ultimately cataract [35]. In addition, proteolysis is another typical characteristic in cataract formation [36–38]. Wang et al proposed that elevated proteolysis resulted from S129R mutation might induce pathology since significant decline of functional proteins changed the protein-protein interaction network [39]. Both in SRA01/04 cells of human and embryonic lens of mouse, DEGs were enriched in proteolysis pathway accompanying with elevated CELF1 expression level, indicating proteolysis might be a crucial pathway regulated by CELF1 in lens development.

In the present study, all five genes enriched in proteolysis were up-regulated in CELF1-OE cells, and four of them were also belong to matrix metalloproteinase family, including MMP1, MMP7, MMP9, and MMP13. The result suggests the potential importance of MMPs in lens development and cataract regulation. In virtually, MMPs are widely distributed in every tissue of the eye under conditions of health and disease, they present a family of proteolytic enzymes that are involved in the breakdown of ECM in normal physiological process, ultimately influencing cell biological activities and morphogenesis [40]. MMP2 and MMP9 were most widely investigated for their important role in cataract. The level of MMP2 and MMP9 activities of patients with steroid induced posterior subcapsular cataract (PSC) in lens epithelial cells (LECs) and the serum was evaluated, MMP2 and MMP9 activities in both LECs and serum were significantly higher in cases with steroid induced PSC [41]. However, MMP9 plays a more important role in mediating TGF-β-induced anterior subcapsular cataract formation than MMP2 [42]. Besides, in human lens epithelial cells, LDL receptor related protein 5 like (LRP5L) may promote angiogenesis by increasing active MMP9. While its mutant, LRP5L-P36R, may inhibit angiogenesis by decreasing active MMP9 and laminin γ1, indicating that LRP5L-P36R may promote the formation of cataract via attenuating biological function of MMP9 [43]. These results suggest the indefinite roles of MMP9 in cataract formation. In this study, we observed that MMP9 was upregulated in both CELF1-OE SRA01/04 cells and mouse E14.5 samples, suggest it might affect lens development or cataract pathogenesis. Based on the definite conclusion that CELF1-KD or CELF1-KO results in defects in lens development or cataractogenesis [9, 20], and its elevated expression level during lens embryonic development [10], we propose that higher CELF1 expression is necessary for lens development and its normal functions, which in turn may protect lens from opacification and cataractogenesis.

Previous study provided evidence that CELF1 associated to the 5’-UTR of human p27 mRNA and suppressed expression of p27 [15]; CELF1 was also inclined to binding with 3’UTR and intron regions of mRNA and further globally changed alternative splicing and translation of multiple genes [12, 31]. Recently, an investigation revealed that Tristeraprolin (TTP) peaks were enriched in CELF1 binding motifs indicating that TTP might cooperate with other RBPs to participate in multiple post-transcriptional process [44]. While, in the current study, we did not find any obvious CELF1 peaks enriched on proteolysis-related genes. Genome-wide chromatin immunoprecipitation method revealed pervasive chromatin-RBP interactions, indicating the transcriptional regulatory ability of RBPs [45]. Based on these existing results, we speculate that CELF1 may regulate expression of proteolysis-related genes at transcriptional level or with an indirectly manner, such as protein-protein interactions. Furthermore, GO analysis showed that CELF1 targeted genes were primarily involved in DNA-dependent transcription regulation, including CDH9, SP4, ZNF420 and ZNF587 that are classical transcriptional factors (TFs). Through binding with DNA, these genes could regulate transcriptional process of many genes [46, 47]. Direct interacting signals between CELF1 and these transcripts were also found from RIP/CLIP sequencing data (Fig. 5A). These CELF1-targeted genes suggest that CELF1 might involve in lens development or cataract pathogenesis by changing expression levels of TFs, which need to be clarified in future. As the CELF1-interacted RNA profiles (RIP or CLIP) were generated from HeLa cells, further investigation and validation experiments are needed to check CELF1 targets, including these TFs, in lens cells.

## Conclusion

A genome-wide profile of CELF1-OE functions in regulating RNA-levels of genes in lens epithelial cells was offered in this study. The expression levels of hundreds of genes regulated by CELF1 were identified, and most of them were linked to multiple biological processes related to lens development. The main definite discovery is that CELF1 may indirectly regulate expression level of MMPs at transcriptional level, suggesting its potential important roles in lens development and cataract formation. Our study contributes to a precise understanding of potential CELF1-targeted therapies in cataract diagnosis and treatment.

## Supplementary Information

**Additional file 1.**

## Data Availability

The datasets generated during and/or analysed during the current study are available in the NCBI Gene Expression Omnibus (GEO) under accession NO. GSE153022.

## Abbreviations

CTSS: Cathepsin S
CELF1: CUG-BP Elav-like family member 1
DEGs: Differentially expressed genes
FPKM: Fragments per kilobase of exon per million fragments mapped
GO: Gene Ontology
GREs: GU-rich elements
KEGG: Kyoto Encyclopedia of Genes and Genomes
MMPs: Matrix metalloproteinase
PCO: Posterior capsule opacification
RBPs: RNA-binding proteins
UTRs: Untranslated regions
