# Supplementary material for: CELF1 promotes matrix metalloproteinases gene expression at transcriptional level in lens epithelial cells
Source: BMC Ophthalmol. 2022 Mar 14;22:122. doi: 10.1186/s12886-022-02344-8 (PMC8922852; doi:10.1186/s12886-022-02344-8)
Supplement: Supplementary file 1 — Additional file 1. [file 12886_2022_2344_MOESM1_ESM.zip › Supplementary Information.docx]

**CELF1 promotes matrix metalloproteinases gene expression at transcriptional level in** **lens epithelial cells**

Jun Xiao^1^, Xi Tian^2^, Siyan Jin^1^, Yanhui He^1^, Meijiao Song^1^, He Zou^1*^

^1^Department of Ophthalmology, The Second Hospital of Jilin University, Changchun city, Jilin province, China.

^2^Department of Pediatrics, The Second Hospital of Jilin University, Changchun city, Jilin province, China.

*Corresponding author: He Zou, E-mail: [15526852075@163.com](mailto:15526852075@163.com)

**Supplementary Information**


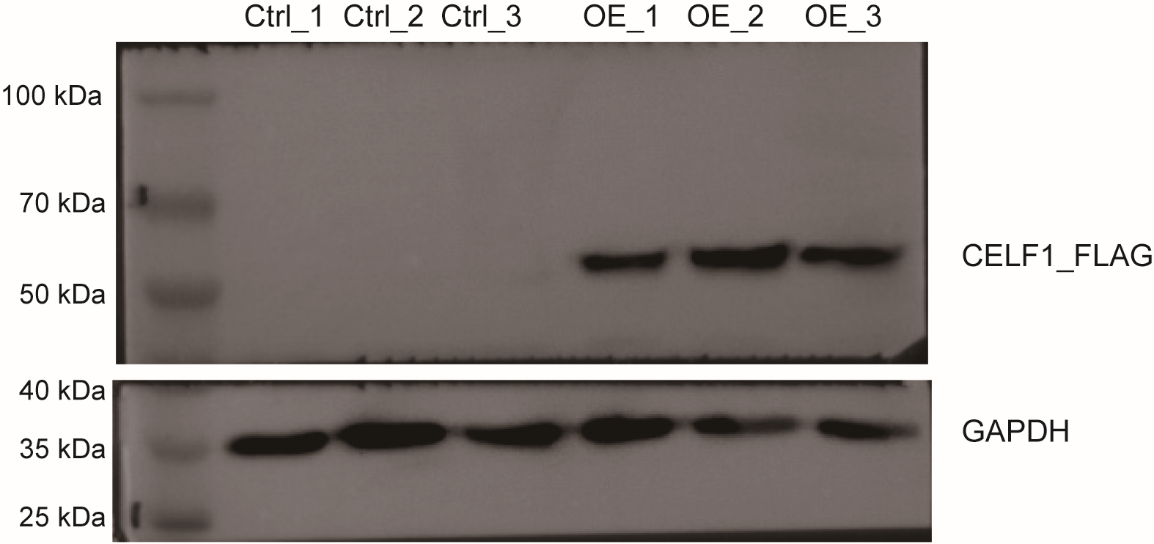


**Fig S1. The original, uncropped gels for WB result to validate the CELF1 overexpression efficiency.**


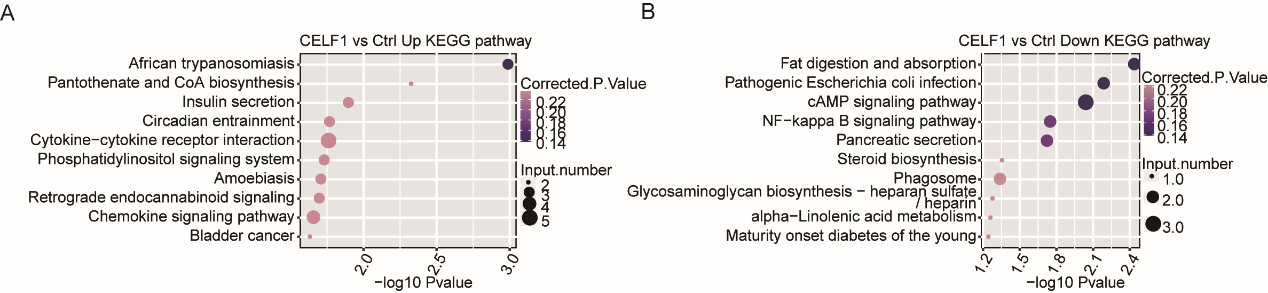


**Fig S2. Bubble plot showing the top ten enriched KEGG pathways for upregulated genes (A) and downregulated genes (B) between CELF1-OE and control samples.**

**Table S1 List of DEGs identified in this study (See attached Excel file).**
