# Supplementary material for: CELF1 promotes matrix metalloproteinases gene expression at transcriptional level in lens epithelial cells
Source: BMC Ophthalmol. 2022 Mar 14;22:122. doi: 10.1186/s12886-022-02344-8 (PMC8922852; doi:10.1186/s12886-022-02344-8)
Supplement: Supplementary file 1 — Additional file 1. [file 12886_2022_2344_MOESM1_ESM.zip › Table S2 letter-probability matrix of CELF1-bound motif.docx]

Table S2. The letter-probability matrix of CELF1-bound motif that was used for motif search in this study.

| Position | probability (A) | probability (C) | probability (G) | probability (U) |
| --- | --- | --- | --- | --- |
| 1 | 0.001 | 0.07 | 0.001 | 0.928 |
| 2 | 0.114 | 0.001 | 0.735 | 0.15 |
| 3 | 0.001 | 0.053 | 0.001 | 0.945 |
| 4 | 0.138 | 0.001 | 0.602 | 0.259 |
| 5 | 0.001 | 0.029 | 0.001 | 0.969 |
| 6 | 0.182 | 0.001 | 0.816 | 0.001 |
| 7 | 0.001 | 0.001 | 0.001 | 0.997 |
