# Supplementary figures and images for: CELF1 promotes matrix metalloproteinases gene expression at transcriptional level in lens epithelial cells
Source: BMC Ophthalmol. 2022 Mar 14;22:122. doi: 10.1186/s12886-022-02344-8 (PMC8922852; doi:10.1186/s12886-022-02344-8)

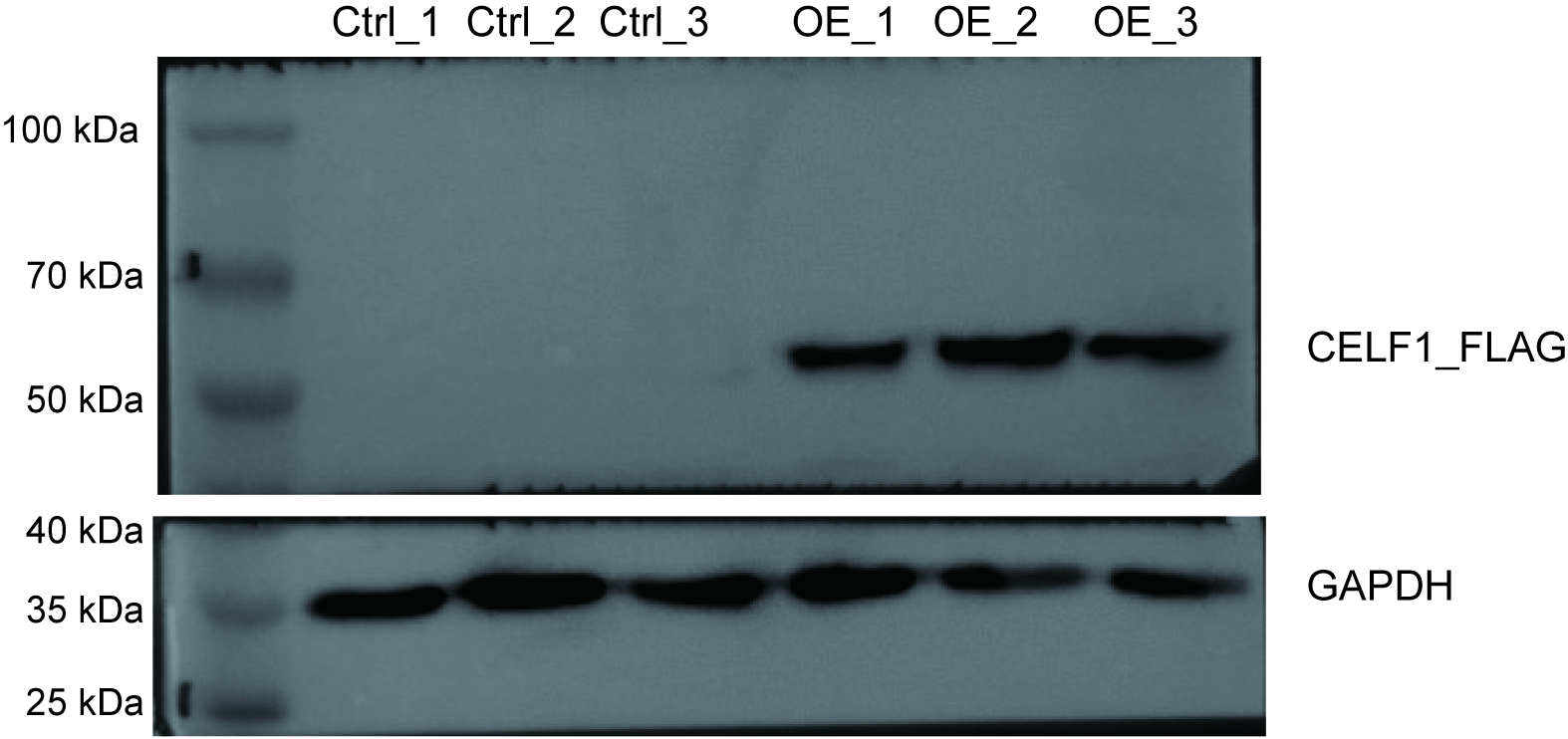

Supplement: Supplementary file 1 — Additional file 1. [file 12886_2022_2344_MOESM1_ESM.zip › FigS1.tif]

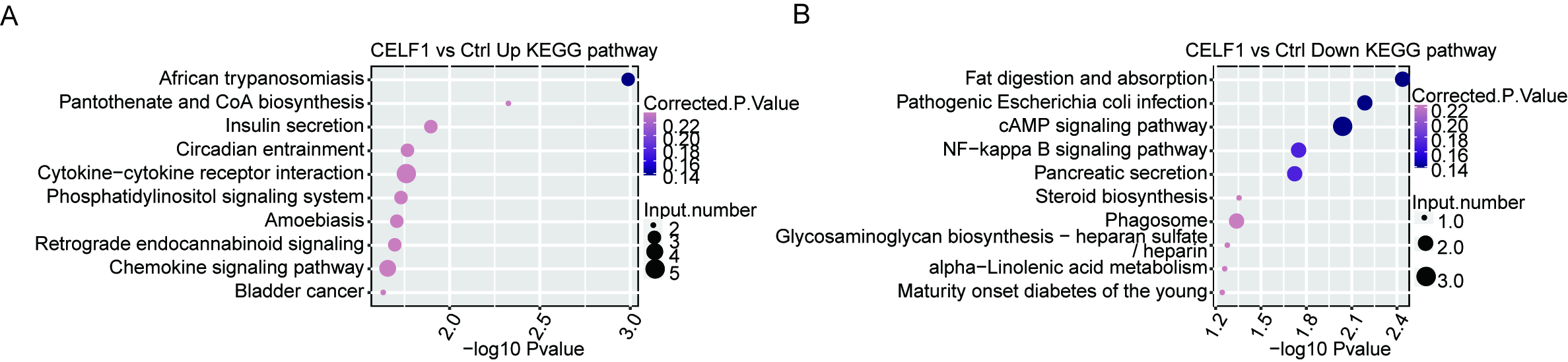

Supplement: Supplementary file 1 — Additional file 1. [file 12886_2022_2344_MOESM1_ESM.zip › FigS2.tif]
